# An artificial protein translation language makes bacteria resistant to viruses

**DOI:** 10.1093/synbio/ysad011

**Published:** 2023-05-27

**Authors:** David M Truong

**Affiliations:** Department of Biomedical Engineering, NYU Tandon School of Engineering, Brooklyn, NY, USA; Department of Pathology, NYU Langone Health, New York, NY, USA

American statesman Benjamin Franklin once stated, ‘… in this world nothing can be said to be certain, except death and taxes’. Had he been a microbiologist, he would have known about another near certainty: that viruses and the organisms they infect, will continue waging cycles of genetic conflict where the host builds immunity against a virus and the virus finds a way around it. But what if we had a way to break that cycle?

In a recent paper by Nyerges *et al.* from George Church’s lab at Harvard Medical School in the USA, the authors engineered the bacterium *Escherichia coli* to be resistant to bacteriophages (bacterial viruses) by using the phage’s reliance on host machinery against them—viruses rely almost entirely on their host’s protein machinery to reproduce, as they cannot replicate on their own (1). To achieve this, they generated a new artificial genetic ‘language’ by which messenger RNAs (mRNAs) are read and translated into proteins. Importantly, the *E. coli* host neither needs nor uses this artificial language to make proteins, but crucially, the bacteriophage does. The system might not only prohibit phage infections but also enable ‘biocontainment’ of the engineered bacteria to prevent escape into the wild.

The genetic code transcribed into mRNAs, which code for proteins, is divided into a three-nucleotide language called codons, which is universal to life as well as viruses. Codons are interpreted by molecules called transfer RNAs (tRNAs), which are bound to specific amino acids. There is redundancy in this language, as there are only 20 amino acids, but 64 possible codons. Because some amino acids have multiple codon/tRNA options, the field of synthetic genomics has long sought to repurpose excess codons for new designer functions, such as inputting non-natural amino acids into proteins (2).

Synthetic genomics practitioners can generate organisms with new designer genomes using advanced DNA synthesis and genetic engineering, and it was a long-standing question whether phage infections could be prevented by recoding entire genomes. Indeed, prior work by the laboratory of Jason Chin at the Medical Research Council Laboratory of Molecular Biology in Cambridge, UK, fully recoded the *E. coli* genome to make the strain Syn61Δ3, in which they genome-wide repurposed two codons specifying serine and one stop sequence (3). In doing so, invading viruses now improperly make proteins, and the same team later concluded that Syn61Δ3 was resistant to viruses (4). The current study by Nyerges *et al.* found instead that Syn61Δ3 was only resistant to some bacteriophages but was susceptible to many other types.

It turns out that many viruses also carry their own tRNAs and thus come ready with the old language when infecting Syn61Δ3. To overcome this loophole, Nyerges focused on changing the tRNAs themselves. They hacked the viruses’ own tRNA–serine pair to now be a tRNA–leucine pair. When they placed this new tRNA into Syn61Δ3 (which no longer has the codons this tRNA recognizes), it caused invading bacteriophages (which do use it) to improperly incorporate leucine instead of serine into their proteins, like typing on a Greek keyboard thinking it was an English one. Using another tRNA, they also addicted Syn61Δ3 to a non-natural amino acid called BipA, thereby preventing its growth in the wild without human help. Concurrent to this, Chin’s group demonstrated a similar strategy, but they instead used weaker *E. coli* tRNAs (5), which the Nyerges study suggests viruses could overcome.

Host organisms like *E. coli* are routinely used for producing biologics and other industrial products. Bacteriophage infections are a common problem in biomanufacturing (6). Genomically recoded organisms like these might prevent virus contamination, saving millions of dollars lost each year. The work also serves as a basis for greater challenges like the Genome-Project Write Consortiums’ goal to make virus-resistant mammalian cell lines (7). If successful, perhaps one day we might be rid of our unwanted relationship with viruses once and for all.
